# Web-Based Public Ratings of General Practitioners in Norway: Validation Study

**DOI:** 10.2196/38932

**Published:** 2023-03-17

**Authors:** Øyvind Bjertnæs, Hilde Hestad Iversen, Rebecka Norman, Jose M Valderas

**Affiliations:** 1 Norwegian Institute of Public Health Oslo Norway; 2 Department of Family Medicine National University Health System Singapore Singapore; 3 Department of Medicine Yong Loo Lin School of Medicine National University of Singapore Singapore Singapore

**Keywords:** web-based rating, questionnaire, psychometric, patient-reported experiences and satisfaction, survey, health care, practitioner, doctor rating, physician rating, patient provider, patient experience, patient satisfaction

## Abstract

**Background:**

Understanding the complex relationships among multiple strategies for gathering users’ perspectives in the evaluation of the performance of services is crucial for the interpretation of user-reported measures.

**Objective:**

The main objectives were to (1) evaluate the psychometric performance of an 11-item web-based questionnaire of ratings of general practitioners (GPs) currently used in Norway (Legelisten.no) and (2) assess the association between web-based and survey-based patient experience indicators.

**Methods:**

We included all published ratings on GPs and practices on Legelisten.no in the period of May 5, 2012, to December 15, 2021 (N=76,521). The questionnaire consists of 1 mandatory item and 10 voluntary items with 5 response categories (1 to 5 stars), alongside an open-ended review question and background variables. Questionnaire dimensionality and internal consistency were assessed with Cronbach α, exploratory factor, and item response theory analyses, and a priori hypotheses were developed for assessing construct validity (chi-square analysis). We calculated Spearman correlations between web-based ratings and reference patient experience indicators based on survey data using the patient experiences with the GP questionnaire (n=5623 respondents for a random sample of 50 GPs).

**Results:**

Web-based raters were predominantly women (n=32,074, 64.0%), in the age range of 20-50 years (n=35,113, 74.6%), and reporting 5 or fewer consultations with the GP each year (n=28,798, 64.5%). Ratings were missing for 18.9% (n=14,500) to 27.4% (n=20,960) of nonmandatory items. A total of 4 of 11 rating items showed a U-shaped distribution, with >60% reporting 5 stars. Factor analysis and internal consistency testing identified 2 rating scales: “GP” (5 items; α=.98) and “practice” (6 items; α=.85). Some associations were not consistent with a priori hypotheses and allowed only partial confirmation of the construct validity of ratings. Item response theory analysis results were adequate for the “practice” scale but not for the “GP” scale, with items with inflated discrimination (>5) distributed over a narrow interval of the scale. The correlations between the web-based ratings GP scale and GP reference indicators ranged from 0.34 (*P*=.021) to 0.44 (*P*=.002), while the correlation between the web-based ratings practice scale and reference indicators ranged from 0.17 (not significant) to 0.49 (*P*<.001). The strongest correlations between web-based and survey scores were found for items measuring practice-related experiences: phone availability (ρ=0.51), waiting time in the office (ρ=0.62), other staff (ρ=0.54-0.58; *P*<.001).

**Conclusions:**

The practice scale of the web-based ratings has adequate psychometric performance, while the GP suffers from important limitations. The associations with survey-based patient experience indicators were accordingly mostly weak to modest. Our study underlines the importance of interpreting web-based ratings with caution and the need to further develop rating sites.

## Introduction

### Background

Patient-centeredness is a core part of health care quality [1,2], but the understanding of the concept varies in primary care, and there are mixed opinions about the relevance of patient-reported data [3,4]. Patient reports are often based on surveys, but reviews of the literature document an increasing focus on patient ratings and reviews from social media and web-based platforms [5-7]. The reviews show a large variation in the organization, content, and setup of such rating sites, including who operates the sites and site rules, the health care level of assessment, the rating questions and rating scale, and the possibilities to write reviews. Rating sites are potentially important for both patients and providers, the former for informing about provider quality and giving the opportunity to provide reviews, and the latter for having access to data for evaluation and improvement. However, a study from the United Kingdom showed that most general practitioners (GPs) had concerns about web-based feedback from patients, questioning the validity and usability of the feedback [8], and another concern is that many patients are not aware of the possibility to rate GPs on the internet [9]. These studies document important obstacles to the use and usefulness of web-based ratings.

An important test of the quality of data on rating sites is to compare ratings with established quality indicators, for example, standardized and scientifically validated survey-based patient experience indicators [10]. Reviews of the literature show a clear association between web-based ratings and survey-based patient experience indicators [5-7] but only identified 1 correlation study in the general practice setting [11]. The UK study found a moderate correlation between survey-based patient experience and web-based ratings, following an analysis of 4950 general practices with ratings on National Health Service (NHS) Choices [11]. However, the median number of ratings for each general practice was 1, which might have seriously affected the correlation estimates. A similar but more recent study from the United Kingdom [12] had larger sample sizes and reported higher correlations. A challenge for both studies is the fact that NHS Choices operates with ratings and reviews at the general practice level without the possibility of rating individual doctors within group practices. Previous research documents substantial variation in patient experiences between individual primary care physicians [13], also within the same practice [14,15], but such differences are masked by systems and surveys conducted or presented at higher health care levels. Thus, the literature on the association between web-based ratings and survey-based patient experience indicators in general practice is weak and further deteriorated by assessing correlations at the practice level, not at the individual GP level.

Legelisten.no is a commercial site established in 2012 where patients have the opportunity to rate and review individual clinicians in Norway, including GPs, gynecologists, chiropractors, dentists, and psychologists. It collects information based on an 11-item questionnaire, but its psychometric properties have not yet been established. A study showed that higher-rated GPs on this site had an increase in demand relative to lower-rated physicians [16]. Legelisten.no is the dominant web-based rating site for health services in Norway, covering all GPs, and with more than 2.6 million unique visitors the last year [17].

### Objectives

Our main objectives of this study are (1) to evaluate the psychometric performance of the web-based questionnaire, including scale-level performance (factor structure, internal consistency, reliability, and known groups validity) and item-level performance (discrimination and difficulty), and (2) to assess the association between web-based scores and survey-based patient experience indicators. If patient web-based ratings are to be used systematically for appraising health care quality and potentially for making decisions about health care–related behavior, they need to be scrutinized by applying rigorous methods. This implies using equivalent quality criteria as for survey-based patient experience indicators, the most important being high-quality instruments and valid indicators at the provider level. The latter is tested by comparing web-based ratings at the GP level to the results of a research-based patient experience survey, that is, a gold standard.

## Methods

### Setting

All residents in Norway are entitled to a regular GP, and around 99% of the population is on a regular GP’s patient list [18]. Norwegian GPs are gatekeepers for the national insurance scheme, and patients are referred from a GP to specialized medical care when needed. The GP practices are, in general, small units. Normally, there are one or more receptionists as well as staff for sampling and analyzing simple tests at the GP practice.

### Web-Based Data

The purpose of the Norwegian rating site (Legelisten.no) is to make it easier for patients to find a well-performing GP or other health professionals included on the site. The web-based questionnaire starts with an open-ended review question about the overall impression of the GP, followed by a mandatory close-ended item about the overall assessment of the treatment with 1 to 5 stars. Each star has a label that emerges when the marker points at it, ranging from “very dissatisfied” to “very satisfied.” The overall assessment part is followed by 10 voluntary evaluation items grouped into (1) accessibility (phone availability, booking time availability, and waiting time in office); (2) trust and communication (trust in advice, trust in insight, listening skills, and enough time); and (3) service (opening hours, other staff, and service facilities). All items have 1 to 5 stars, but the labels for the stars vary: all trust and communication stars range from “no, not at all” to “yes, absolutely”; the service stars range from “very dissatisfied” to “very satisfied”; while the accessibility stars are adjusted to the relevant time span (seconds or minutes for telephone, minutes for waiting time in office, and days for consultation booking time). The rating questions are included in Multimedia Appendix 1. The questionnaire also includes self-reported variables about age, gender, and the number of yearly consultations with the GP. The instrument used on Legelisten.no was developed by the company itself but lacks documentation of its development, reliability, and validity. All published ratings and reviews of GPs at Legelisten.no in the period May 26, 2012, to December 15, 2021, were included in this study.

### Survey Data

The Norwegian Institute of Public Health conducted a national patient experience survey with GPs in 2021, with 10 patients randomly selected from each of a random sample of 2000 GPs (N=20,000). The sample consisted of patients aged 16 years and older with at least one contact with the GP in the last 12 months. Patients registered in a national digital portal received a digital invitation to the survey with an electronic response option, while the others were mailed a postal invitation letter with an electronic response option. Two reminders were sent to nonrespondents, both including a pen-and-paper questionnaire and an electronic response option. The patient experiences with GP questionnaire (PEQ-GP) consists of 5 scales with 18 items [19]: assessment of the GP (8 items), coordination (2 items), patient enablement (3 items), accessibility (2 items), and practice (3 items). All items had a 5-point response format ranging from 1 (not at all) to 5 (to a very large extent).

To obtain robust estimates at the GP level, we randomly selected 50 GPs from the main sample, and 290 additional patients from these GPs, or all if the number of patients was below 290.

### Statistical Analysis

Web-based items were assessed for missing data [14] and ceiling effects [20]. We performed classical psychometric tests, including exploratory factor analysis and an assessment of internal consistency reliability. Exploratory factor analysis was used to assess the underlying structure of the items (principal axis factoring, Promax rotation, and factors with eigenvalue above 1), while internal consistency reliability was used to assess if items adequately contribute to the scale construct (item-total correlation, Cronbach α, and Cronbach α if an item is deleted). We calculated scale scores for respondents with a valid response for at least half of the items on a scale. Known groups validity is an aspect of construct validity [21]. No single observation can prove construct validity. Instead, multiple tests are conducted simultaneously, and construct validity is supported if all or most tests are going in the expected direction. Known groups validity was assessed by testing the association between scale scores and 3 background questions about the patient (age, gender, and the number of yearly consultations) using the chi-square test for gender and the Mantel-Haenszel test for the trend of age and the number of consultations. Based on the results of the former national survey in Norway [22], we hypothesized that increasing the number of consultations would be positively associated with patient experiences, and age would not be associated with patient experiences except for the practice scale, while women overall would report better experiences than men. The graded response model was applied for polytomous items in item response theory (IRT) analysis for each scale separately and evaluated item performance in terms of item discrimination (higher means better) and item category location (threshold separation for scale coverage), that is, difficulty [23,24].

Web-based ratings at the GP level were correlated with patient-reported experience scores from the survey subsample of all GPs that had both web-based ratings and survey estimates using all scales and items from the web-based data and all scales and similar items from the survey data. Prior to the correlation analysis, exploratory factor analysis and internal consistency testing were conducted on the PEQ-GP in the survey subsample to verify the scale structure from the original validation [19]. The Spearman rank correlation coefficient was used in the correlation analysis. We hypothesized that scales and items measuring the same construct would have the strongest correlations; for example, GP scales or items from the survey would have stronger correlations with web-based GP scales or items than with web-based practice scales or items. All analyses were conducted with SPSS (version 26.0; IBM Corp), except for IRT analysis, where we used R (version 3.6.3; R Foundation for Statistical Computing; package *mirt*).

### Ethical Considerations

The study was part of the Norwegian Institute of Public Health program for patient experience surveys with the GP and the GP office (2021-2025), which is based on an approved Data Protection Impact Assessment and an approval from the Health Directorate.

## Results

Of the web-based raters (N=76,521), 65.5% (n=50,122) reported gender, 61.5% (n=47,086) reported age, and 61.3% (n=46,897) reported the number of yearly visits to the GP. Of those reporting background variables, 64.0% (n=32,074) were women, 74.6% (n=35,113) were in the age range of 20-50 years, and 64.5% (n=28,798) reported having 5 or fewer consultations with the GP each year. Among women, 80.0% (n=25,659) of the raters were in the 20-50 years age group, while the corresponding figure for men was 65.3% (n=11,785). Compared to the gender and age distribution in the national survey, women and patients in the age group of 20-40 years were heavily overrepresented in the web-based sample (Table 1).

The number of items missing varied from 18.9 to 27.4 for the voluntary items (Table 2). In total, 7 of the 11 evaluation items were heavily skewed toward the positive end of the scale, with >50% ticking the most positive response category. In total, 4 of 11 items had a U-shaped distribution, with the 2 largest percentages being the extreme values, that is, 1 or 5 stars. A total of 3 items about the practice level had the lowest scores on the 5-point scale, with the mean score being 2.9 (SD 1.38) for phone availability, 3.3 (SD 1.40) for booking time availability, and 3.5 (SD 1.23) for waiting time in the office.

Factor analysis identified 2 scales with eigenvalues above 1, explaining 73.7% of the variation of the observed variables (Table 3). The factors were labeled “GP” (5 items) and “practice” (6 items) and had Cronbach α values of .98 and .85, respectively.

**Figure 1:**
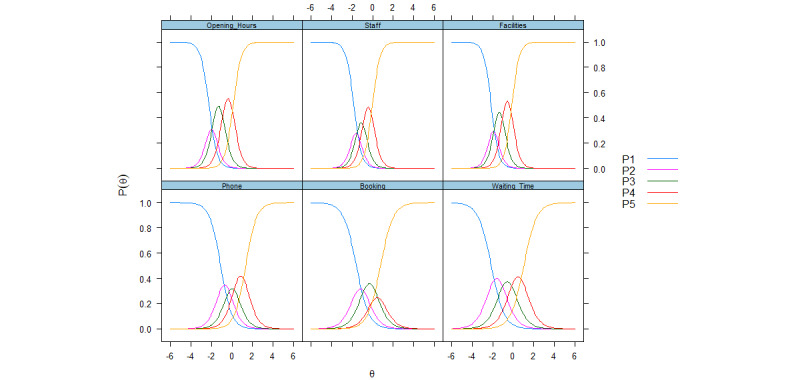
Categorical response curves for web-based practice scale items. P is the probability of endorsing a response category.

**Figure 2:**
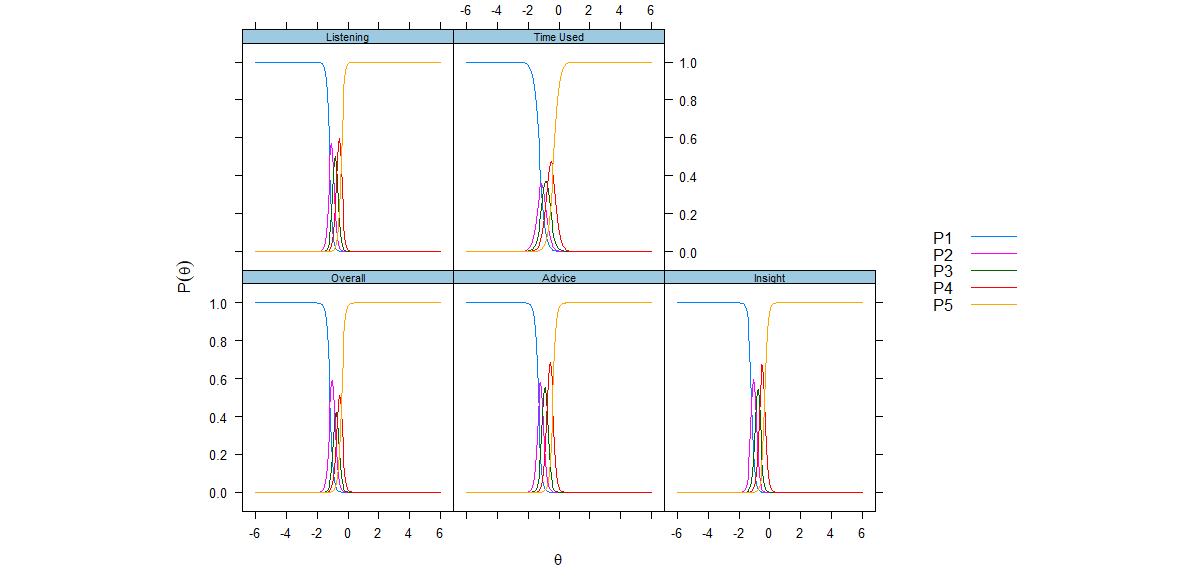
Categorical response curves for web-based GP scale items. P is the probability of endorsing a response category.

Tests of construct validity showed that men had significantly better experiences than women, while age and the number of consultations were positively associated with patient experiences for both scales (Table 4). Item results from IRT analysis were adequate for the practice scale (Table 5), with discrimination values ranging from 1.56 (waiting time in office) to 2.74 (service facilities). Thresholds for the practice items covered θ values below and above 0, except for the items about other staff and service facilities, where the highest threshold (b4) was lower than 0. The categorical response curve visualizes item discrimination and item category thresholds (Figure 1) and further shows that the second response category has questionable value for several of the practice items (opening hours, staff, facilities, and booking), while the fourth response category also seems to underperform for the item on booking. Response categories seemed to be well-ordered for all items on the GP scale, which notwithstanding consistently showed inflated discrimination (>5) and covered a narrow interval below the middle of the scale: threshold b4 ranged from −0.44 (listening skills) to −0.32 (trust insight). The categorical response curve for the GP scale visualizes the high discrimination for the items and shows that response categories 2 to 4 had limited value (Figure 2).

The response rate in the survey subsample was 41.4% (n=5623), with response rates at the GP level ranging from 20.3% (n=58) to 58.5% (n=172). Psychometric testing of the PEQ-GP in the subsample verified the original scale structure: GP (Cronbach α=.93), coordination (Cronbach α=.89), patient enablement (Cronbach α=.91), accessibility (Cronbach α=.76), and practice (Cronbach α=.87). The mean number of responses for each GP in the survey subsample was 119.6 (SD 58-172), compared to 13.7 (SD 1-73) ratings for the same GPs in the web-based data. In total, 24 GPs had fewer than 10 web-based ratings. Correlations at the GP level were conducted for the 46 of 50 GPs that had both web-based ratings and survey estimates (Table 6). Significant correlations were mostly found between web-based data and survey data for scales or items with similar content, and the strongest correlations were found for concrete items measuring the practice level: phone availability (Spearman ρ=0.51), waiting time in the office (Spearman ρ=0.62), and other staff (Spearman ρ=0.54-0.58). The web-based GP scale correlated significantly with all survey scales and items measuring the GP, with correlations varying from 0.35 to 0.44. The web-based practice scale correlated significantly with the accessibility scale and similar items forming the web-based practice scale (Spearman ρ=0.36-0.49), but not with the survey practice scale (Spearman ρ=0.17). The overall web-based rating was correlated with all GP scales and items, varying from 0.37 to 0.45 (0.38 for the overall survey item), and only significantly correlated with 1 practice item (phone availability).

**Table 1:** Background variables for web-based sample and national surveys.

Characteristics		Web-based sample^a^ (N=76,521)	National survey^b^ (N=18,860)
**Gender,** **n (%)**			
	Men	18,048 (36.0)	8503 (45.1)
	Women	32,074 (64.0)	10,357 (54.9)
**Age (years),** **n (%)**			
	Below 20	863 (1.8)	804 (4.3)
	20-30	13,866 (29.4)	2788 (14.8)
	31-40	12,133 (25.8)	2969 (15.7)
	41-50	9114 (19.4)	2902 (15.4)
	51-60	6147 (13.1)	3071 (16.3)
	>60	4963 (10.5)	6326 (33.5)
**Yearly visits to GP^c^, n (%)**			
	0	504 (1.1)	—^d^
	1-2	12,039 (25.7)	—
	3-5	16,255 (34.7)	—
	6-10	11,252 (24)	—
	11-20	5058 (10.8)	—
	>20	1789 (3.8)	—

^a^Voluntary self-reported variables: 50,122 reported gender, 47,086 reported age, and 46,897 reported yearly visits.

^b^Register-based variables for total sample in national survey.

^c^GP: general practitioner.

^d^Not available.

**Table 2:** Item descriptives for web-based patient evaluations from 2012 to 2021 (N=76,521).

	Missing, n (%)	1 star, n (%)^a^	2 stars, n (%)	3 stars, n (%)	4 stars, n (%)	5 stars, n (%)^a^	Mean (SD)	Median (IQR)
Overall rating^b^	—^c^	12,429 (16.2)	5677 (7.4)	3093 (4.0)	4860 (6.4)	50,462 (65.9)	4.0 (1.57)	5 (3-5)
Phone availability	19,989 (26.1)	12,115 (21.4)	10,996 (19.5)	11,816 (20.9)	12,453 (22.0)	9152 (16.2)	2.9 (1.38)	3 (2-4)
Booking time availability	18,632 (24.3)	8264 (14.3)	8918 (15.4)	14,247 (24.6)	9551 (16.5)	16,909 (29.2)	3.3 (1.40)	3 (2-5)
Waiting time in office	18,410 (24.1)	4651 (8.0)	8831 (15.2)	14,326 (24.7)	16,453 (28.3)	13,850 (23.8)	3.5 (1.23)	4 (3-4)
Trust in advice	14,500 (18.9)	6597 (10.6)	4207 (6.8)	3741 (6.0)	6249 (10.1)	41,227 (66.5)	4.2 (1.39)	5 (4-5)
Trust insight	15,337 (20.0)	8556 (14.0)	4077 (6.7)	3131 (5.1)	6376 (10.4)	39,044 (63.8)	4.0 (1.49)	5 (3-5)
Listening skills	14,491 (18.9)	8514 (13.7)	3922 (6.3)	2835 (4.6)	4392 (7.1)	42,367 (68.3)	4.1 (1.49)	5 (4-5)
Enough time	15,040 (19.7)	7799 (10.2)	3735 (6.1)	3874 (6.3)	6877 (11.2)	39,196 (63.8)	4.1 (1.44)	5 (3-5)
Opening hours	20,960 (27.4)	2105 (3.8)	2229 (4.0)	8375 (15.1)	16,984 (30.6)	25,868 (46.6)	4.1 (1.05)	4 (4-5)
Other staff	20,546 (26.9)	3898 (7.0)	3058 (5.5)	6643 (11.9)	13,884 (24.8)	28,492 (50.9)	4.1 (1.21)	5 (4-5)
Service facilities	20,627 (27.0)	2311 (4.1)	2048 (3.7)	6662 (11.9)	14,384 (25.7)	30,489 (54.5)	4.2 (1.06)	5 (4-5)

^a^One star also represents floor effect, while 5 stars represent ceiling effect.

^b^Overall rating is mandatory on Legelisten.no, while the other rating variables are voluntary. Question formulations and response categories are shown in Multimedia Appendix 1.

^c^Not available.

**Table 3:** Factor loadings and internal consistency reliability for web-based items or scales.

		Factor analysis^a^		Internal consistency reliability		
		Factor 1	Factor 2	Item-total correlation	Cronbach α	Cronbach α if item deleted
**GP^b^ scale**				N/A^c^	.982	N/A
	Overall rating	0.917		0.955	N/A	.976
	Trust in advice	0.950		0.946	N/A	.978
	Trust insight	0.963		0.955	N/A	.976
	Listening skills	0.997		0.960	N/A	.976
	Enough time	0.871		0.918	N/A	.982
**Practice scale**				N/A	.848	N/A
	Phone availability		0.686	0.630	N/A	.825
	Booking time availability		0.620	0.604	N/A	.831
	Waiting time in office		0.550	0.600	N/A	.829
	Opening hours		0.709	0.670	N/A	.819
	Other staff		0.796	0.662	N/A	.817
	Service facilities		0.706	0.662	N/A	.820

^a^Factor analysis with listwise deletion (pairwise deletion and imputation of means for missing values gave the same solution). Values below 0.2 are not shown. Eigenvalues: factor 1: 6.65; factor 2: 1.45.

^b^GP: general practitioner.

^c^N/A: not applicable.

**Table 4:** Associations between self-reported background variables and web-based scales^a^.

Background variables		GP^b^ scale		Practice scale	
		Mean (SD)	*P* value	Mean (SD)	*P* value
**Gender**			.007		<.001
	Men	80.2 (32.9)		69.6 (23.8)	
	Women	79.3 (34.2)		67.5 (23.2)	
**Age (years)**			<.001		<.001
	<20	72.3 (38.4)		65.3 (24.3)	
	20-30	75.4 (36.7)		65.5 (24.6)	
	31-40	79.6 (33.6)		67.6 (23.7)	
	41-50	82.5 (31.4)		69.8 (22.6)	
	51-60	85.5 (28.7)		71.9 (21.2)	
	>60	89.3 (23.7)		74.6 (19.6)	
**Number of consultations each year**			<.001		<.001
	0	54.7 (43.5)		55.9 (29.7)	
	1-2	77.0 (35.0)		65.8 (24.1)	
	3-5	80.9 (32.3)		68.4 (23.0)	
	6-10	81.5 (32.2)		69.4 (22.3)	
	11-20	83.3 (31.6)		71.1 (22.2)	
	>20	83.2 (32.3)		72.6 (23.4)	

^a^Missing was 0% for the GP scale and 18.8% for the practice scale.

^b^GP: general practitioner.

**Table 5:** Parameter estimates from item response theory analysis of the web-based scales^a^.

			a		b1		b2		b3		b4
**GP^b^ scale**											
	Overall rating	9.25		−1.17		−0.87		−0.68		−0.43	
	Trust in advice	9.03		−1.34		−1.04		−0.76		−0.39	
	Trust insight	9.87		−1.18		−0.91		−0.66		−0.32	
	Listening skills	10.04		−1.20		−0.94		−0.72		−0.44	
	Enough time	5.57		−1.25		−0.98		−0.70		−0.33	
**Practice scale**											
	Phone availability	1.87		−1.10		−0.33		0.37		1.33	
	Booking time availability	1.62		−1.58		−0.77		0.15		0.77	
	Waiting time in office	1.56		−2.13		−1.07		−0.08		1.03	
	Opening hours	2.51		−2.25		−1.74		−0.88		0.11	
	Other staff	2.65		−1.82		−1.39		−0.83		−0.03	
	Service facilities	2.74		−2.14		−1.70		−1.00		−0.14	

^a^Separate item response theory analysis for each scale. Graded response model. a: discrimination; b1-b4: thresholds.

^b^GP: general practitioner.

**Table 6:** Associations^a^ at the GP^b^ level between web-based scores (all scales and items) and survey-based scores (all scales and items with similar content).

		Patient experience indicators (survey)													
		Scales						Items							
Web-based ratings		GP	Enablement	Coordination	Practice	Accessibility	Overall		GP insight	GP time	GP interest	Wait office	Phone	Wait acute	Wait elective
**GP (scale)**		*0.41* ^c^	*0.42*	*0.34*	0.20	0.25	*0.35*		*0.44*	*0.39*	*0.39*	0.22	*0.36*	0.22	0.10
	Overall rating	*0.41*	*0.43*	*0.37*	0.27	0.25	*0.38*		*0.45*	*0.39*	*0.40*	0.24	*0.39*	0.26	0.15
	Trust in advice	*0.30*	0.28	0.17	0.12	0.14	0.24		*0.32*	0.27	0.29	0.21	0.24	0.10	0.08
	Trust insight	*0.34*	*0.35*	0.21	0.11	0.15	0.28		*0.40*	*0.30*	*0.32*	0.24	0.28	0.10	0.09
	Listening skills	*0.32*	*0.31*	0.18	0.05	0.14	0.27		*0.33*	*0.30*	*0.31*	*0.31*	*0.28*	0.10	0.10
	Enough time	*0.32*	*0.32*	0.23	0.08	0.09	*0.25*		*0.34*	*0.31*	*0.30*	0.22	0.24	0.07	0.30
**Practice (scale)**		0.09	0.17	0.06	0.17	*0.45*	0.11		0.16	0.13	0.07	*0.49*	*0.37*	*0.36*	*0.45*
	Phone availability	0.03	0.13	0.05	0.24	0.27	−0.00		0.09	0.11	0.04	*0.41*	*0.51*	0.20	0.27
	Booking time availability	−0.11	0.02	−0.08	−0.00	*0.34*	−0.11		0.00	−0.03	−0.11	*0.34*	0.19	0.21	*0.41*
	Waiting time in office	0.06	0.08	0.02	0.09	*0.31*	0.06		0.09	0.14	0.02	*0.62*	*0.39*	0.24	*0.30*
	Opening hours	0.08	0.22	0.07	0.04	0.24	0.09		0.21	0.03	0.09	0.10	0.16	0.18	0.24
	Other staff	−0.03	0.04	0.03	*0.31*	*0.54*	0.06		0.00	0.02	−0.04	*0.34*	*0.32*	*0.43*	*0.58*
	Service facilities	0.19	0.30	0.19	0.22	*0.33*	0.26		−0.18	0.17	0.18	−0.18	0.23	0.25	*0.31*

^a^Spearman ρ. Of 50 randomly selected GPs, a total of 46 had both web-based ratings and survey-based indicators and are included here.

^b^GP: general practitioner.

^c^Values in italics denote significant correlations.

## Discussion

### Principal Findings

The web-based practice scale had adequate psychometric results, while the GP scale performed poorly. The associations with survey-based indicators were mostly weak to modest.

The Norwegian rating site allows ratings of individual GPs and includes detailed evaluations of the GP and the practice through an 11-item questionnaire. This kind of specific evaluation is lacking in other web-based rating systems for general practice, for example, NHS Choices in the United Kingdom [12]. The use of an 11-item questionnaire gave the opportunity to conduct psychometric testing of the instrument using standard tests for patient-experience instruments [25]. To our knowledge, this is the first study in the general practice setting to conduct psychometric analysis of web-based ratings. Other sites, like NHS Choices, only have one or a few overall rating items, thereby limiting opportunities for this level of scrutiny. While factor analysis and internal consistency testing performed well and identified 2 scales (GP and practice), tests of construct validity were mixed, with some results in the opposite of the expected direction (eg, for gender), and IRT results for the GP scale showed poor performance, including high discrimination and narrow scale coverage. The discrimination values imply a violation of the local independence assumption in IRT with excess covariation between items [24], while the narrow scale coverage follows from the U-shaped distribution for most GP items, which have also been found elsewhere [12]. Furthermore, correlations between web-based scores and survey-based scores were significant but low to modest for the GP scale or items, but higher for items with more concrete evaluations of the practice. Reviews of the literature show a clear association between web-based ratings and survey-based patient experience indicators [5-7], and 2 studies in the general practice setting showed small to moderate correlations [11,12]. However, having providers with few raters negatively affects the correlation level [10], and in our subsample, a total of 24 (49%) of the GPs had fewer than 10 ratings. The problem with the small number of raters was the same in the UK studies in general practice using NHS Choices, with the median number of ratings for each practice varying from 1 in 2009-2010 [11] to 17 from 2009-2016 [12]. The former included all GP practices in the United Kingdom, while the latter included practices from one clinical commissioning group in England. It seems like rating sites should not only develop, test, and implement strategies for increasing the number of ratings at the practice or GP level but also clearly communicate uncertainty and consider a lower limit for the number of raters before providing quantitative scores (eg, a minimum of 10). Furthermore, the U-shaped distributions mean that average values have little value [12], and instead the percentage above or below certain thresholds could be used. All in all, allowing evaluations at the GP level and using a multiitem questionnaire are potentially useful, but this potential is currently not being fulfilled in the Norwegian rating site, as indicated by poor measurement properties for several GP scale criteria and only weak to modest correlations with survey-based patient experience scores.

The inherent positivity bias in satisfaction measurement was obvious for all GP items [26], with as much as 64%-68% of all raters choosing the most positive response category. This is much higher than the ceiling effects for GP items in the national patient experience surveys [19,22] and negatively affects the possibility of identifying differences over time and between GPs. Beyond initiatives to include more persons with nonextreme evaluations, there seems to be a need for developing and testing approaches to reduce the ceiling effect. A previous study in the hospital setting showed that almost half of the comments from patients with excellent ratings of health services (ie, top scores) were about negative or mixed experiences [27]. A study in the general practice setting found fewer negative experiences in the top-box group, but more than 35% of patients selecting the best or second-best response option described mixed or negative experiences [12]. One possible approach to reduce ceiling effects is unbalanced response scales, that is, using more positive than negative response categories and dividing the positive category into different degrees of positive. A previous study showed that an unbalanced response scale reduced the ceiling effect [28], but whether this also differentiates between current top-scoring patients should be assessed. Another approach is to further use free text comments from patients by applying machine learning to automatically conduct sentiment analysis and create quantitative variables from these analyses [29]. All ratings at Legelisten.no demand a written review with at least 100 characters (50 previously), which means that top-scoring patients might be differentiated based on the sentiment of the review. In addition to these more research-based initiatives, simple adjustments could be considered, for example, formulating more concrete questions about experiences with the GP and changing the order or presentation of questions.

### Limitations

The response rate in the patient experience survey was just above 40% (n=5623), raising concern about the generalizability and ability to function as a gold standard for the web-based data. The response rate was comparable to or higher than that of other national surveys, for example, the General Practice Patient Survey in the United Kingdom [11], but more important than response rate is nonresponse bias. Previous follow-up studies of nonrespondents in patient experience surveys have shown small differences between respondents in the ordinary data collection and respondents in the follow-up study [30,31], which at least indicate a lesser concern related to nonresponse bias. Another limitation is the inability to compare web-based ratings with clinical quality indicators, which follows from the fact that the Norwegian quality indicator system lacks quality indicators at the GP and practice level. At least from a clinical perspective, it would be useful to assess such associations, but we argue that survey-based indicators and web-based data are even more relevant to compare given that both aim to measure patient-centeredness. Previous research shows that correlations between clinical quality indicators and web-based ratings are lower than those between web-based ratings and survey-based patient experience indicators [6,11]. Another limitation is that we included data from a significant period of time. A potential downside could be changes in policies or systems in the broad health care landscape, which could bias responses over the period. The current low number of ratings for each GP means that there are not enough data to disaggregate results and assess consistency in web-based ratings over time. Finally, the study would have benefited from a direct comparison of results obtained from a survey format of the same questionnaire. This was not possible in our study but is a possible avenue for future research.

### Conclusions

Evaluations at the GP level with the 11-item questionnaire would have been potentially useful. This potential is far from being realized, as evidenced by poor measurement properties according to multiple criteria for the GP scale and only weak to modest correlations with the survey-based patient experience indicators. The web-based questionnaire should be further improved, refined, and validated, and the presentation of results should be informed by the metric performance of the questionnaire. Rating sites should develop, test, and implement strategies for increasing the number of ratings, including how to secure responses from persons with nonextreme evaluations, communicate more clearly statistical uncertainty, and consider a lower limit for the number of raters before providing quantitative scores.

To realize the extreme potential of web-based rating sites, the validity and reliability of the underlying measurement tools need to be established, and the communication of results needs to more clearly report statistical uncertainty due to the metric performance of the tools themselves and biases and limitations in sampling.

## Appendix

Multimedia Appendix 1 Web-based rating questions at Legelisten.no.

## Abbreviations

GP: general practitioner
IRT: item response theory
NHS: National Health Service
PEQ-GP: patient experiences with GP questionnaire

## Data Availability

The Norwegian Institute of Public Health data set is available from the corresponding author on reasonable request, while access to web-based data requires an approval from Legelisten.no.
