# Supplementary material for: Web-Based Public Ratings of General Practitioners in Norway: Validation Study
Source: JMIR Form Res. 2023 Mar 17;7:e38932. doi: 10.2196/38932 (PMC10131642; doi:10.2196/38932)
Supplement: Multimedia Appendix 1 [file formative_v7i1e38932_app1.pdf]

Multimedia Appendix 1. Web-based rating questions at Legelisten.no

1. Overall assessment – how satisfied are you with the treatment? 1 star: very dissatisfied; 2 stars: dissatisfied; 3 stars: neither/nor; 4 stars: satisfied; 5 stars: very satisfied.
2. Telephone queue – how long do you normally have to wait in telephone queue when booking an appointment? 1 star: more than 5 minutes; 2 stars: 2-5 minutes; 3 stars: 1-2 minutes; 4 stars: 10-60 seconds; 5 stars: less than 10 seconds.
3. Booking an appointment – how quick do you normally get an appointment? 1 star: more than 10 days; 2 stars: 5-10 days; 3 stars: 2-5 days; 4 stars: next day; 5 stars: same day.
4. Wating time after scheduled time – how long do you normally have to wait before the consultation start? 1 star: more than 10 days; 2 stars: 5-10 days; 3 stars: 2-5 days; 4 stars: next day; 5 stars: same day.
5. Advice and recommendations – do you trust that advice and recommendations about treatment are in your/the patients best interest? 1 star: no, not at all; 2 stars: to a little extent; 3 stars: to some extent; 4 stars: to a large extent; 5 stars: yes, absolutely.
6. Help to insight – do you feel that you are helped in understanding the disease/condition? 1 star: no, not at all; 2 stars: to a little extent; 3 stars: to some extent; 4 stars: to a large extent; 5 stars: yes, absolutely.
7. Ability to listen – do you feel that you are listened to and receive answer to your questions? 1 star: no, not at all; 2 stars: to a little extent; 3 stars: to some extent; 4 stars: to a large extent; 5 stars: yes, absolutely.
8. Time use – do you feel that the time used for you is sufficient? 1 star: no, not at all; 2 stars: to a little extent; 3 stars: to some extent; 4 stars: to a large extent; 5 stars: yes, absolutely.
9. Opening hours – how satisfied are you with the opening hours? 1 star: very dissatisfied; 2 stars: dissatisfied; 3 stars: neither/nor; 4 stars: satisfied; 5 stars: very satisfied.
10. Other staff – how satisfied are you with the other staff? (courtesy, kindness, efficiency). 1 star: very dissatisfied; 2 stars: dissatisfied; 3 stars: neither/nor; 4 stars: satisfied; 5 stars: very satisfied.
11. The clinic – how satisfied are you with other aspects of the clinic/service? (cleaning, waiting room, location, other). 1 star: very dissatisfied; 2 stars: dissatisfied; 3 stars: neither/nor; 4 stars: satisfied; 5 stars: very satisfied.
